# Editorial: *Ad-Hoc* Selection of Lactic Acid Bacteria for Non-conventional Food Matrices Fermentations: Agri-Food Perspectives

**DOI:** 10.3389/fmicb.2021.681830

**Published:** 2021-05-07

**Authors:** Erica Pontonio, Carlo Giuseppe Rizzello

**Affiliations:** ^1^Department of Soil, Plant and Food Science, University of Bari Aldo Moro, Bari, Italy; ^2^Department of Environmental Biology, “Sapienza” University of Rome, Rome, Italy

**Keywords:** lactic acid bacteria, fermentation, microbial selection, bioactive compounds, non- conventional food matrices

Consumers demand for health-related food products and the need to differentiate products aiming at addressing the most pressing economic, environmental and ethical issues are forcing the researchers to focus their activities on the design of innovative foods. The employment of non-conventional matrices (e.g., pulses, single-cell protein, bee-pollen and tropical fruits) as novel food ingredients and the use of selected starters (mainly lactic acid bacteria) to enhance their nutritional and functional features through fermentation, are both considered as promising scientific approaches (Canon et al.; Niccolai et al.; Pontonio et al.; Abe Sato et al.; Filannino et al.). Alternative strategies like the multi-copy sod-expressing cassettes and the use of specific nutrients (e.g., glycerol) were proposed to enhance the antioxidant and antimicrobial potential as well as the process performances of selected starters (Kong et al.; Zhang et al.).

The increasing demand for dairy-free food and beverage products with high nutritional quality and the nutrition recommendations of the Health Authorities in terms of protein, fibers and bioactive compounds, led to the optimization of lactose-free plant-based products (Niccolai et al.; Pontonio et al.). Legumes and *Arthrospira platensis* were used as protein sources and selected lactic acid bacteria *Lactiplantibacillus plantarum* and *Levilactobacillus brevis* as tool to improve the protein digestibility and antioxidant potential (Niccolai et al.; Pontonio et al.) while decreasing the anti-nutritional factors (Pontonio et al.). The presence of probiotics and their survival rate during storage are an added value for novel functional lactose-free beverages (Niccolai et al.; Pontonio et al.).

Formulating foods and beverages combining animal and plant supplies seems also to be a valuable approach to differentiate the market offer of sustainable products (Canon et al.). However, in order to increase the added value of these products while decreasing the drawbacks of such raw materials (e.g., carbohydrates responsible for malabsorption and intolerance and digestive discomfort) an *ad-hoc* selection of strains with specific functions and their assembly into co-cultures preparation might be required (Canon et al.).

Fermentation can further improve matrices already characterized by high functional potential. Bee bread and bee-collected pollen are recognized as valuable dietary supplements for human nutrition however, the latter needs to be processed before the human consumption to increase the availability of nutrients for intestinal absorption (Filannino et al.). The role of fermentation with selected *Apilactobacillus kunkeei* strains and *Hanseniaspora uvarum* in the improvement of the anti-inflammatory and immuno-modulatory features of bee-collected pollen has been demonstrated and attributed to the greater bio-accessibility of inherent bioactive compounds (Filannino et al.).

According to the target of a specific food production, the potentiality of the microbial strains intended to be used as starters can be tailored. For example, Kong et al. proposed a specific strategy to improve the oxidative stress resistance in *Streptococcus thermophilus* and consequently the survival in food matrices in which it could be used as starter. Moreover, the study of specific genetic traits positively affecting the microorganism growth rate, or the release of bioactive compounds is considered of great importance (Zhang et al.). In this context, the role of glycerol for growth enhancement and reuterin formation in *Limosilactobacillus reuteri* has been investigated and novel insights about the ecology of *L. reuteri* with biotechnological and therapeutic relevance were highlighted (Zhang et al.).

The rising consumers awareness as well as the pandemic issue have “fast-tracked” the consumer interest toward the health-related role of the diet, and the benefits of ingredients and specific functional foods. Among the functional foods, those providing probiotics benefits have been largely investigated by the scientific community.

Many factors need to be considered during the screening of potential probiotic strain, however the safety evaluation, resistance to low pH and bile acids as well as antimicrobial activity are considered of main importance. Beside the potential role on the intestinal microbiota balance, the antimicrobial activity is moreover considered a useful control tool toward spoilage and pathogenic microorganisms in foods, especially in non-Western Countries, which often face safety issues related to food contamination.

Abe Sato et al. screened and isolated potential probiotic lactic acid bacteria from the açai fruits aiming at producing a fermented probiotic juice in which the microbial starter might inhibit the growth of pathogens often responsible for food intoxication (Abe Sato et al.). Several strains showed some common characteristics of probiotics and antagonistic activity against pathogens thus suggesting that açai fruits are a potential source of lactic acid bacteria with interesting probiotic characteristics although complementary tests need to be carried out (Abe Sato et al.).

Overall, the studies collected in this journal topic demonstrated that different non-conventional food matrices are suitable substrates, when adequately bioprocessed, to produce innovative functional foods. Among all, the lactic acid bacteria fermentation seems to be a valuable tool to enhance both the nutritional and functional features of the raw matrices, however the effects of fermentation are strongly dependent on the selected microbial starters. Hence, an *ad-hoc* selection of suitable strains and an in-depth investigation of their physiological and genotypic characteristics are a key point of the process.

## Author Contributions

EP and CR contributed equally to the proposal, editorial work of this Research Topic, and equally contributed to the writing of the Editorial.

## Conflict of Interest

The authors declare that the research was conducted in the absence of any commercial or financial relationships that could be construed as a potential conflict of interest.

